# Agreement between software programmes of body composition analyses on abdominal computed tomography scans of obese adults

**DOI:** 10.20945/2359-3997000000174

**Published:** 2019-09-25

**Authors:** Erica Roberta Barbalho, Ilanna Marques Gomes da Rocha, Galtieri Otávio Cunha de Medeiros, Rogerio Friedman, Ana Paula Trussardi Fayh

**Affiliations:** 1 Programa de Pós-Graduação em Educação Física Centro de Ciências da Saúde Universidade Federal do Rio Grande do Norte Natal RN Brasil Programa de Pós-Graduação em Educação Física, Centro de Ciências da Saúde, Universidade Federal do Rio Grande do Norte, Natal, RN, Brasil; 2 Programa de Pós-Graduação em Nutrição Centro de Ciências da Saúde Universidade Federal do Rio Grande do Norte Natal RN Brasil Programa de Pós-Graduação em Nutrição, Centro de Ciências da Saúde, Universidade Federal do Rio Grande do Norte, Natal, RN, Brasil; 3 Hospital Universitário Onofre Lopes Universidade Federal do Rio Grande do Norte Natal RN Brasil Hospital Universitário Onofre Lopes, Universidade Federal do Rio Grande do Norte, Natal, RN, Brasil; 4 Universidade Federal do Rio Grande do Sul Porto Alegre RS Brasil Unidade Endócrina, Universidade Federal do Rio Grande do Sul, Porto Alegre, RS, Brasil

**Keywords:** Body composition, skeletal muscle mass, subcutaneous adipose tissue, visceral fat, imaging techniques

## Abstract

**Objective:**

A large number of studies have used abdominal computed tomography (CT) to quantify body composition, and different software programmes have been used to perform these analyses. Thus, this comparison is important to enable researchers to know the performance of more accessible software.

**Subjects and methods:**

Fifty-four abdominal CT scans of obese (BMI 30 to 39.9 kg/m^2^), sedentary adults (24-41 years) patients from a Brazilian single center were selected. Two software programs were compared: Slice-O-Matic (Tomovision, Canada) version 5.0 and OsiriX version 5.8.5. The body composition analysis were segmented using standard Hounsfield unit (HU) (adipose tissue: -190 to +30 and skeletal muscle: -29 to +150) and measured at the mid third lumbar vertebra (L3) level on a slice showing both transversal processes. Bland-Altman limits of agreement analyses were used to assess the level of agreement between Slice-O-Matic and OsiriX.

**Results:**

A total of fifty-four participants were evaluated, with majority women (69%), mean of age 31.3 (SD 6.5) years and obesity grade I most prevalent (74.1%). The agreement, in Bland-Altman analysis, between Slice-O-Matic and OsiriX analisys for the muscle mass tissue, visceral adipose tissue and subcutaneous adipose tissue were excellent (≥ 0.954) with P-values < 0.001.

**Conclusion:**

These findings show that Slice-O-Matic and OsiriX softwares agreement in measurements of skeletal muscle and adipose tissue and sarcopenia diagnosis in obese patients, suggesting good applicability in studies with body composition in this population and clinical practice.

## INTRODUCTION

Obesity can be defined as a pathologic accumulation of body fat (1). It is associated with increased cardiovascular morbidity and mortality, due to a wide spectrum of prevalent metabolic, inflammatory and clotting abnormalities that can accelerate the process of atherosclerosis (2,3). Obesity is one of the most common diseases on a global scale. In 2010, overweight and obesity were estimated to cause 3.4 million deaths, 3.9% of years of life lost, and 3.8% of disability-adjusted life-years worldwide (4). Although clinically defined as a Body Mass Index (BMI) ≥ 30 kg/m^2^ (5), a better characterization of obesity can be made evaluating body composition, especially body fat and muscle mass (6).

Among different adipose tissues, the visceral adipose tissue (VAT) has been recognized as the most related to several clinical and laboratoristic parameters of cardiovascular disease risk and the metabolic syndrome (7). Subcutaneous adipose tissue (SAT) does not appear to have the pro-inflammatory activity of VAT, but some studies did find an association between SAT – especially at abdominal level – and insulin resistance (IR) (8). So, over the last few decades, the evaluation and differential quantification of specific adipose tissue compartments in the body has gained paramount importance. Computed tomography (CT) and magnetic resonance imaging (MRI) are considered to be the gold standard for measuring different adipose tissues, as well as skeletal muscle mass (9). The widespread use of these techniques, particularly MRI, is limited by their high cost, low availability, time requirements, and, for CT, X-ray exposure (10).

A large number of studies have used abdominal CT to quantify body composition parameters in a variety of clinical situations, such as renal (11) and hepatic (12,13) disease, obesity (3) and cancer (14-16). Different software programmes have been used to perform these body composition analyses. It has been previously found that the software system used to analyze CT images can affect data analysis and interpretation (17). In the available literature, different software programs have been used to analyze body composition images by CT, such as Slice-O-Matic, Fat Seg, OsiriX (18) and NIH ImageJ (17). It is important that the comparability of these various software programmes should be known. However, only very few studies have looked into this comparison, and the different authors assessed different clinical situations and populations. It is known that body composition is influenced by health status and ethnicity (6); therefore, studies in specific populations may contribute to the comparison and validation of these software packages. Therefore, the aim of this study was to investigate the agreement of two different software packages for the assessment of body composition, specially cross-sectional skeletal muscle and subcutaneous and visceral adipose tissue measurements on abdominal CT scans of obese individuals. Currently, Slice-O-Matic is the most used software for body composition analyses of CT scans, but your price is upper than all others commercially available softwares, as OsiriX. Thus, this comparison is important to enable researchers to know the performance of more accessible software.

## SUBJECTS AND METHODS

### Subjects

Fifty-four abdominal CT scans of patients from a Brazilian single center – Hospital de Clínicas de Porto Alegre (Porto Alegre, RS, Brazil) – obtained between 2009 and 2011 were selected. Data for sample characterization were collected directly by researchers. All patients were obese adults (BMI 30 to 39.9 kg/m^2^), of both sexes, aged 22-41 years, sedentary, and not taking any drugs. The present study was conducted in accordance with the Declaration of Helsinki, and all procedures involving human subjects/patients were approved by the Ethics Committee of the *Hospital de Clínicas de Porto Alegre* (08-282). Written informed consent was obtained from all subjects.

### Anthropometric evaluation

Anthropometric measurements for sample characterization included body mass, height, waist and hip circumference. Height was measured with a ﬁxed stadiometer (Tonelli Ltda., Brazil), with a 1 mm precision. Body weight was measured on a digital scale (MEA-03200; Plenna, Brazil) in light indoor clothes, without shoes. Waist circumference was measured with an inelastic tape measure (Sanny, Brazil), halfway between the last rib and the iliac crest. The nutritional status was classiﬁed by the BMI (kg/m^2^), according to standard cutoffs (5).

### CT image analysis protocol – Skeletal muscle and adipose tissue area measurements

The SAT and VAT areas (cm^2^) were measured at the mid third lumbar vertebra (L3) level, on a single slice showing both transversal processes. The cross-sectional muscle area measurements included the following muscles: psoas, paraspinal, transverse abdominal, external oblique, internal oblique, and rectus abdominis. All abdominal CT scans were assessed on identical slices in a random order by three trained observers (G.O., I.M.G.R and E.R.B.), with great expertise on radiological anatomy and extensive experience in skeletal muscle and adipose tissue area measurements using software programmes. The observers were blinded for each other’s measurements and for patient details.

Two software programmes were compared: Slice-O-Matic (Tomovision, Canada) and OsiriX. The CSMA, VAT, and SAT were segmented using standard Hounsfield unit (HU) thresholds in both software programmes. An intensity window between -30 and +150HU was used for skeletal muscle tissue. For adipose tissue, an intensity window between -190 and -30 HU was used.

### SliceOmatic analysis protocol

Slice-O-Matic (Tomovision, Canada) version 5.0 was used. Tissue was semi-automatically selected with the ‘Region Growing’ mode using the ‘Grow 2D’ and ‘Paint’ tools. Non-skeletal muscle tissue regions adjacent to skeletal muscle having radiological density between the predefined HU thresholds were manually erased using the ‘Paint’ tool. Cutaneous tissue was included in the SAT measurement. A 3.2 GHz Intel^®^ Core^TM^ i5 Dell personal computer was used.

### OsiriX analyses protocol

The open-source 32-bit edition of OsiriX version 5.8.5 was used. The ‘Grow Region (2D/3D Segmentation)’ tool was used to semi-automatically select skeletal muscle and adipose tissue regions within the chosen HU intensity thresholds. Non-skeletal muscle tissue regions adjacent to skeletal muscle were manually removed from the area selection using the brush option. The brush option was also used to manually erase intraluminal areas with contents having radiological densities between -190 and 30HU, resembling fat content. Cutaneous tissue was not included in the SAT measurement. The skeletal muscle and adipose tissue areas were computed automatically and expressed in square centimeter using a 1.3GHz Intel^®^ Core™ i5 MacBook Air (Apple Inc., Cupertino, CA, USA) computer.

### Statistical analysis

The Shapiro-Wilk test was used to verify data normality. Normally distributed data that were presented as mean and standard deviation. Data that were not normally distributed were expressed as median and interquartile amplitude. Independent samples *t*-test was used to compare differences between genders. Paired samples t-test (for normally distributed data) or Wilcoxon’s signed rank test were used to compare differences between the different software packages’ results. Intra-class correlation coefficient (ICCs – two-way mixed single measures model and absolute agreement) with 95% confidence interval was used to verify inter-software agreement for the cross-sectional skeletal muscle estimation. The ICCs were interpreted as poor (0.00-0.49), fair to good (0.50-0.74), and excellent (0.75-1.00), as proposed by Shrout and Fleiss. Finally, Bland-Altman limits of agreement analyses were used to assess the level of agreement between Slice-O-Matic and OsiriX. All statistical analyses were carried out on SPSS version 21.0 for Windows (Statistical Package for Social Sciences, Chicago, IL, USA). The Jaccard similarity coefﬁcient was used to verify the inter-software similarity of measurements (19). Initially, an overlap of two measurements was created by the calculation of the index of dissimilarity, according to formula:

$$D\;=\;\frac{1}{2}\;\sum_{i=1}^{N}\;\left|\frac{ai}{A}\;-\;\frac{bi}{B}\right|$$,

where, *‘ai’* and *‘bi’* are each value of variables measures (i.e. each value of SliceOmatic and OsiriX), and ‘A’ e ‘B’ are the sum of all values of each variable (i.e sum of values of SliceOmatic and sum of values of OsiriX). Finally, the Jaccard similarity coefﬁcient was defined as 1 – D (index of dissimilarity). A Jaccard similarity coefﬁcient of 1 represents perfect overlap of two samples, whereas 0 represents no overlap.

## RESULTS

Table 1 shows the anthropometric characterization of the sample. The majority of the sample were women (69%), with grade I obesity (74.1%). In line with the prevalence of obesity, high mean values were found for abdominal circumference, waist circumference and hip circumference, with differences between genders.

**Table 1:** Clinical and anthropometric patients characteristics (n = 54)

Variables	Mean ± SD	
Age (years)	31.3 ± 6.5	
Weight (kg)	94.3 ± 17.1	
Height (m)	1.66 ± 0.1	
Body Mass Index (kg/m^2^)	33.9 ± 3.7	

	**Male (n = 17)**	**Female (n = 37)**

Abdominal circumference (cm)	112.6 ± 8.9	108.5 ± 6.6
Waist circumference (cm)	107.7 ± 7.4	96.1 ± 6.2*
Hip circumference (cm)	116.9 ± 6.2	121.4 ± 5.4
Waist-to-hip ratio (a.u.)	0.92 ± 0.05	0.79 ± 0.05*

* Paired t test (p < 0.05).

**Figure 1:**
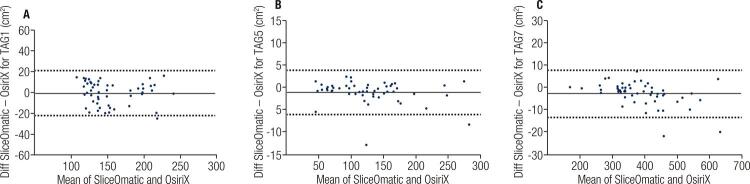
Bland-Altman 95% limits of agreement plots for the agreement between the SliceOmatic and OsiriX analysis for MMT (Panel A), VAT (Panel B), and SAT (Panel C). The continuous line is the mean of the difference and the dotted lines are the 95% limits of agreement.

The agreements between Slice-O-Matic and OsiriX analisys for muscle tissue mass, visceral adipose tissue and subcutaneous adipose tissue were excellent (≥ 0.954) with P-values < 0.001. Additionally, the mean Jaccard similarity coefficients for the inter-software were closely the perfect (Table 2). Figure 1 shows the Bland-Altman 95% agreement plots, with the mean difference and 95% limits of agreement for the MMT, VAT, and SAT 7 between Slice-O-Matic and OsiriX analysis. The limits of agreement were -22.3 to 21.3 for MMT, -6.1 to 3.9 for VAT, and -13.2 to 7.9 for SAT. In all plots, the limits include the mean difference between Slice-O-Matic and OsiriX analyses for all variables analyzed.

**Table 2:** Mean cross-sectional skeletal muscle and adipose tissue measurements on abdominal and inter-software agreement (n=54)

Variables	SliceOmatic	OsiriX	Mean difference (95% CI)	P-value	ICC (95% CI)	Mean Jaccard Index (range)
MMT (cm^2^)	155.2 ± 35.9 (112.3; 239.8)	155.7 ± 36.8 (100.2; 241.0)	-0.5 (-3.6; 2.6)	0.967^b^	0.954 (0.921 – 0.973)	0.990 (0.984-0.995)
VAT (cm^2^)	127.0 ± 56.8 (42.4; 277.9)	128.0 ± 57.4 (44.6; 286.2)	-1.1 (-1.8; -0.4)	0.004^a^	0.999 (0.998 – 0.999)	0.990 (0.978-0.997)
SAT (cm^2^)	388.3 ± 94.6 (171.0; 630.6)	391.0 ± 97.0 (170.8; 644.5)	-2.7 (-4.1; -1.2)	0.001^b^	0.998 (0.996 – 0.999)	0.990 (0.984-0.996)

Data expressed as mean ± standard deviation (minimum and maximum values); CI, confidence interval; ICC, Intra-class correlation coefficient. ^a^ paired sample t-test. ^b^ Wilcoxon’s signed rank test.

MMT: muscle mass tissue; VAT: visceral adiposity tissue; SAT: subcutaneous adiposity tissue.

Regarding the classification of low muscularity (MMT), there was an inter-software agreement (Cohen’s k) of 1.00 between Slice-O-Matic and OsiriX (*P* < 0.001). There was no difference in classification of individuals with and without sarcopenia between software programmes. According to the cut-off values used, Slice-O-Matic and OsiriX analysis diagnosed sarcopenia in the same 6 individuals (11.1%). All individuals that presented sarcopenia were male.

## DISCUSSION

Currently, CT is considered one of the most accurate methods for analyzing quantitative and qualitative changes in body composition, especially when investigating skeletal muscle mass and distinguishing adipose tissue in different body compartments (12,20). A dedicated computer software is required to quantify and determine components in different tissues. In our study, we found a strong correlation of muscle mass, visceral adipose tissue and subcutaneous adipose tissue as evaluated by Slice-O-Matic and OsiriX. To date, this is the second study to compare these softwares. Previously, van Vugt and cols. (18) had compared both these and other software packages, with similar results to our. However, they evaluated the performance of software in cancer patients, which have different body composition from the population evaluated in the present study. Irving and cols. (17) also observed good agreement between Slice-O-Matic and NIH Image J for analysis of adipose tissue and skeletal muscle mass, but they did not evaluate OsiriX. Thus, it is important to compare the performance of these software in different clinical situations and populations, since these characteristics can impact the body composition.

Since Mourtzakis and cols. (21) demonstrated that cross-sectional areas of fat and fat-free tissues at lumbar level measured on CT scanning correlate strongly with whole-body tissues, innumerous studies have been published with CT images of abdominal area quantifying body composition. The technique has found a potential clinical application in oncology, for research suggests that the technique may help identify patients at higher risk for chemoterapy toxicity (14,22-26) based on higher percentages of VAT or muscle mass depletion. Other diseases may benefit from the study of body composition by imaging. In obese persons, this may especially important, because of the many contributions of adipose tissue to the disease. Evaluation and quantification of adipose tissue are fundamental in the field of body composition. Understanding body composition is crucial for understanding human metabolism and its alterations (27).

CT imaging has potential methodological limitations and patient-related limitations. An important patient-related limitation is the presence of ascites/end stage of liver diseases (28). Fluid in the abdominal cavity confounds the reading of Hounsfield units for each component of the body composition in Slice-O-Matic software, leading to an underestimation of adipose tissue. The main methodological limitation is the potential intra- and inter-observer variability. Lack of standardization, or careless conducting of the image obtaining procedures (with consequent loss of accuracy) must be minimized by thorough operator’s training and the observance of a proper fasting period before the examination.

In the present study, was observed significant differences in VAT and SAT measurements between softwares. This could be due to the greater complexity of the measurement technique for VAT, that needs to be manually erased. In relation to SAT analysis, the difference could partly been explained by the fact that in OsiriX the cutaneous adipose tissue in not included in the SAT, in contrast to Slice-O-Matic (18). Due to the fact that every tissue of interest needs to be manually selected in OsiriX, in contrast to the other programmes in which methods of delineating or a painting brush can be used to select regions of interest, the use of OsiriX may produce different results in the analysis of body composition.

Since BMI has been shown to be an imprecise measurement of fat free mass and fat mass, body composition analysis with CT images presents great practical significance for some disease, especially when physicians routinely use CT for diagnosis and follow-up of their patients. However, the heterogeneity of the populations evaluated, as well as the use of different softwares and scanning sites hampers the very definition of a “gold standard”.

It is important to highlight the drawbacks of the CT technique for the evaluation of body composition. CT is an expensive technique for the health system. The patient is exposed to X-ray radiation. It is estimated that 1.5%-2.0% of all cancers in the United States may be attributable to radiation from CT studies (29). Nevertheless, the exposure in this technique is only a fraction of the usual, since a single slice is used. Either software demands expertise in its use. Slice-O-Matic is more user-friendly, but it is not freely distributed and a paid, relatively expensive license is required. For a setting of limited funding like the Brazilian health system, this may represent a decisive characteristic. Additionally, these softwares present limitations about the operating systems: OsiriX is only compatible with Macintosh, while Slice-O-Matic is only compatible with Windows.

In conclusion, the findings suggest that the use of either software (Slice-O-Matic and OsiriX) package should to assess changes body composition. Thus, the decision about the use may be based on characteristics of each health system and the presence of technical expertise.

## References

[1] Kelly T, Yang W, Chen CS, Reynolds K, He J. Global burden of obesity in 2005 and projections to 2030. Int J Obes. 2008;32(9):1431-7. 10.1038/ijo.2008.102 18607383

[2] Fayh APT, Reischak-Oliveira A, Friedman R. Insulin resistance and endothelial dysfunction in obesity: Impact of diet and physical training. Rev Bras Med. 2015;72(3):6P.

[3] Fayh APT, Lopes AL, Fernandes PR, Reischak-Oliveira A, Friedman R. Impact of weight loss with or without exercise on abdominal fat and insulin resistance in obese individuals: A randomised clinical trial. Br J Nutr. 2013;110(3):486-92. 10.1017/S0007114512005442 23302544

[4] World Health Organization. Obesity and overweight [Internet]. 2014 [cited 2017 Jul 4]. Available from: http://www.who.int/mediacentre/factsheets/fs311/en/.

[5] World Health Organization. Physical status: the use and interpretation of anthropometry. Report of a WHO Expert Committee. Vol. 854, World Health Organization technical report series. 1995. p. 1-452. 8594834

[6] Andreoli A, Garaci F, Cafarelli FP, Guglielmi G. Body composition in clinical practice. Eur J Radiol. 2016;85(8):1461-8. 10.1016/j.ejrad.2016.02.005 26971404

[7] Roriz AKC, Passos LCS, De Oliveira CC, Eickemberg M, Moreira PDA, Sampaio LR. Evaluation of the accuracy of anthropometric clinical indicators of visceral fat in adults and elderly. PLoS One. 2014;9(7):5-10. 10.1371/journal.pone.0103499 PMC4117503 25078454

[8] Patel P, Abate N. Role of subcutaneous adipose tissue in the pathogenesis of insulin resistance. J Obes. 2013;2013:489187. 10.1155/2013/489187 PMC3649613 23691287

[9] Yip C, Dinkel C, Mahajan A, Siddique M, Cook GJR, Goh V. Imaging body composition in cancer patients: visceral obesity, sarcopenia and sarcopenic obesity may impact on clinical outcome. Insights Imaging. 2015;6(4):489-97. 10.1007/s13244-015-0414-0 PMC4519815 26070723

[10] Mazzoccoli G. Body composition: Where and when. Eur J Radiol [Internet]. 2016;85(8):1456-60. Available from: 10.1016/j.ejrad.2015.10.020. 26564096

[11] Nguyen GK, Mellnick VM, Yim AK-Y, Salter A, Ippolito JE. Synergy of Sex Differences in Visceral Fat Measured with CT and Tumor Metabolism Helps Predict Overall Survival in Patients with Renal Cell Carcinoma. Radiology. 2018;287(3):171504. 10.1148/radiol.2018171504 29558292

[12] Benjamin J, Shasthry V, Kaal CR, Anand L, Bhardwaj A, Pandit V, et al. Characterization of body composition and definition of sarcopenia in patients with alcoholic cirrhosis: A computed tomography based study. Liver Int. 2017;37(11):1668-74. 10.1111/liv.13509 29065258

[13] Fujiwara N, Nakagawa H, Kudo Y, Tateishi R, Taguri M, Watadani T, et al. Sarcopenia, intramuscular fat deposition, and visceral adiposity independently predict the outcomes of hepatocellular carcinoma. J Hepatol. 2015;63(1):131-40. 10.1016/j.jhep.2015.02.031 25724366

[14] Cushen SJ, Power DG, Murphy KP, McDermott R, Griffin BT, Lim M, et al. Impact of body composition parameters on clinical outcomes in patients with metastatic castrate-resistant prostate cancer treated with docetaxel. Clin Nutr ESPEN. 2016;13:e39-45. 10.1016/j.clnesp.2016.04.001 28531567

[15] Deluche E, Leobon S, Desport JC, Venat-Bouvet L, Usseglio J, Tubiana-Mathieu N. Impact of body composition on outcome in patients with early breast cancer. Support Care Cancer. 2017;26(3):861-8. 10.1007/s00520-017-3902-6 PMC5785600 28948392

[16] Daly LE, Bhuachalla EBN, Power DG, Cushen SJ, James K, Ryan AM. Loss of skeletal muscle during systemic chemotherapy is prognostic of poor survival in patients with foregut cancer. J Cachexia Sarcopenia Muscle. 2018;9(2):315-25. 10.1002/jcsm.12267 PMC5879982 29318756

[17] Irving BA, Weltman JY, Brock DW, Davis CK, Gaesser GA, Weltman A. NIH ImageJ and Slice-O-Matic computed tomography imaging software to quantify soft tissue. Obesity. 2007;15(2):370-6. 10.1038/oby.2007.573 17299110

[18] van Vugt JLA, Levolger S, Gharbharan A, Koek M, Niessen WJ, Burger JWA, et al. A comparative study of software programmes for cross-sectional skeletal muscle and adipose tissue measurements on abdominal computed tomography scans of rectal cancer patients. J Cachexia Sarcopenia Muscle. 2017;8(2):285-97. 10.1002/jcsm.12158 PMC5697014 27897414

[19] Jaccard P. Étude comparative de la distribution ﬂorale dans une portion des alpes et des jura. Bull Soc Vaud Sci Nat. 1901;37:547-79.

[20] Sergi G, Trevisan C, Veronese N, Lucato P, Manzato E. Imaging of sarcopenia. Eur J Radiol. 2016;85(8):1519-24. 10.1016/j.ejrad.2016.04.009 27117135

[21] Mourtzakis M, Prado CMM, Lieffers JR, Reiman T, McCargar LJ, Baracos VE. A practical and precise approach to quantification of body composition in cancer patients using computed tomography images acquired during routine care. Appl Physiol Nutr Metab. 2008;33(5):997-1006. 10.1139/H08-075 18923576

[22] Anandavadivelan P, Brismar TB, Nilsson M, Johar AM, Martin L. Sarcopenic obesity: A probable risk factor for dose limiting toxicity during neo-adjuvant chemotherapy in oesophageal cancer patients. Clin Nutr. 2016;35(3):724-30. 10.1016/j.clnu.2015.05.011 26065721

[23] Tan BHL, Brammer K, Randhawa N, Welch NT, Parsons SL, James EJ, et al. Sarcopenia is associated with toxicity in patients undergoing neo-adjuvant chemotherapy for oesophago-gastric cancer. Eur J Surg Oncol. 2015;41(3):333-8. 10.1016/j.ejso.2014.11.040 25498359

[24] Morley JE, Abbatecola AM, Argiles JM, Baracos V, Bauer J, Bhasin S, et al. Sarcopenia With Limited Mobility: An International Consensus. HHS Public Heal. 2011;12(6):403-9. 10.1016/j.jamda.2011.04.014 PMC5100674 21640657

[25] Ryan AM, Power DG, Daly L, Cushen SJ, Bhuachalla EN, Prado CM. Cancer-associated malnutrition, cachexia and sarcopenia: the skeleton in the hospital closet 40 years later. Proc Nutr Soc. 2016;75:199-211. 10.1017/S002966511500419X 26786393

[26] Marty E, Liu Y, Samuel A, Or O, Lane J. A review of sarcopenia: Enhancing awareness of an increasingly prevalent disease. Bone. 2017;105:276-86. 10.1016/j.bone.2017.09.008 28931495

[27] Bazzocchi A, Filonzi G, Ponti F, Amadori M, Sassi C, Salizzoni E, et al. The Role of Ultrasonography in the Evaluation of Abdominal Fat: Analysis of Technical and Methodological Issues. Acad Radiol. 2013;20(10):1278-85. 10.1016/j.acra.2013.07.009 24029060

[28] Cruz RJJ, Dew MA, Myaskovsky L, Goodpaster B, Fox K, Fontes P, et al. Objective Radiological Assessment of Body Composition in Patients with End-Stage Liver Disease: Going Beyond the BMI Ruy. Transplantation. 2015;95(4):617-22. 10.1097/TP.0b013e31827a0f27 PMC4504696 23348896

[29] Brenner DJ, Hall EJ. Computed tomography–an increasing source ofradiation exposure. N Engl J Med. 2007;357:2277-84. 10.1056/NEJMra072149 18046031

